# Additional Considerations for US Residency Selection After Pass/Fail USMLE Step 1. Comment on “The US Residency Selection Process After the United States Medical Licensing Examination Step 1 Pass/Fail Change: Overview for Applicants and Educators”

**DOI:** 10.2196/47763

**Published:** 2023-08-17

**Authors:** Yacine Sow, Ameya Gangal, Howa Yeung, Travis Blalock, Benjamin Stoff

**Affiliations:** 1 Morehouse School of Medicine Atlanta, GA United States; 2 Department of Dermatology Emory University School of Medicine Atlanta, GA United States

**Keywords:** admission, assessment, postgraduate training, selection, standardized testing, USMLE, medical school, medical students, residency application, research training

As medical students navigating the new landscape of residency selection after the switch to a pass/fail USMLE (United States Medical Licensing Examination) Step 1, we read a recent viewpoint by Ozair et al [1] with great interest. We hope to offer a unique perspective and present additional potential solutions for residency programs and medical schools to consider.

We agree with the authors’ observation that research productivity is now necessary for a successful match with competitive specialties. Ozair et al [1] discussed the disadvantages for international medical graduates (IMGs) and provided a cost-benefit analysis of students trying to maximize research output. We would add that research by medical students relies on access to well-funded research institutions and adequate mentorship. This impacts IMGs as well as students attending institutions without home residency programs [2]. To gain access to research experiences, medical students increasingly undertake research years [3]. These research fellowships, some of which are paid whereas others are not, are competitive and limited. Unpaid research fellowships pose several problems, such as potential loss of student status and subsequent requirement for loan repayments, loss of health insurance, and need to fund living expenses and relocation costs [3]. Students with already-limited access to research experiences can face prohibitively high financial burdens in this context.

As Ozair et al [1] provided recommendations and resources for residency applicants, we would like to offer recommendations for residency programs. To mitigate inequity surrounding research metrics, programs may consider offering year-long, paid fellowships for students without home programs, IMGs, and students with financial needs. Programs can also promote a collaborative environment through dedicated outreach, research funding, and away rotations for students at programs with less research funding. Access to research opportunities should be especially considered as part of a holistic review.

In light of the barriers to engaging in research, Ozair et al [1] suggested securing protected research time for a competitive match. Given the barriers to acquiring year-long research fellowships, we suggest medical school curricula allocate time for research and networking experiences to explore fields of interest and bolster applications. The authors also remarked that exam-related anxiety is likely to increase, as candidates now only have one chance to obtain a top score on Step 2 [1]. Thus, we suggest medical schools allocate a 6- to 8-week dedicated period for students to prepare for Step 2 or allow students to take Step 2 before completing all third-year clerkships. The Wake Forest School of Medicine employed an abbreviated set of “core” clerkships where students took Step 2 halfway through their third year, providing more time to complete the remaining electives and prepare for the residency application process [4].

The shift to binary Step 1 grading resulted from good intentions but has had unintended consequences, particularly for medical students. Thus, we hope residency programs and medical schools support an equitable residency application process; provide transparency about methods of assessing applicants, including those related to research output; and make curricular adaptions to support students during this time of transition.

## Abbreviations

IMG: international medical graduate
USMLE: United States Medical Licensing Examination
